# Noncanonical NF-κB signaling and the essential kinase NIK modulate crucial features associated with eosinophilic esophagitis pathogenesis

**DOI:** 10.1242/dmm.030767

**Published:** 2017-12-01

**Authors:** Kristin Eden, Daniel E. Rothschild, Dylan K. McDaniel, Bettina Heid, Irving C. Allen

**Affiliations:** 1 Department of Biomedical Sciences and Pathobiology, Virginia Maryland College of Veterinary Medicine, Virginia Tech, Blacksburg, VA 24060, USA; 2 Department of Biomedical Science, Virginia Tech Carilion School of Medicine, Roanoke, VA 24016, USA

**Keywords:** EoE, Eosinophils, Inflammation, TSLP, Gastrointestinal, NF-κB-inducing kinase

## Abstract

Eosinophilic esophagitis (EoE) is an allergic disease of the esophagus driven by T cell and eosinophil responses to dietary allergens, resulting in chronic mucosal inflammation. Few spontaneous animal models of esophageal eosinophilia exist, with most studies relying on artificial sensitization procedures. NF-κB-inducing kinase (NIK; MAP3K14) is a key signaling molecule of the noncanonical NF-κB (NFKB1) pathway, an alternative signaling cascade producing chemokines involved in lymphoid stroma development and leukocyte trafficking. *Nik*^−/−^ mice have been shown to develop a hypereosinophilic syndrome in peripheral blood and major filtering organs; however, the gastrointestinal mucosa of these mice has not been well characterized. We show that *Nik*^−/−^ mice develop significant, localized eosinophilic esophagitis that mimics human EoE, including features such as severe eosinophil accumulation, degranulation, mucosal thickening, fibrosis and basal cell hyperplasia. The remainder of the GI tract, including the caudal stomach, small intestine and colon, in mice with active EoE are unaffected, also similar to human patients. Gene expression patterns in esophageal tissue of *Nik^−/−^* mice mimics human EoE, with thymic stromal lymphopoetin (TSLP) in particular also elevated at the protein level. In gene expression data sets from human biopsy specimens, we further show that many genes associated with noncanonical NF-κB signaling are significantly dysregulated in EoE patients, most notably a paradoxical upregulation of NIK itself with concurrent upregulation of powerful protein-level destabilizers of NIK. These findings suggest that *Nik^−/−^* mice could be useful as a spontaneous model of specific features of EoE and highlight a novel role for noncanonical NF-κB signaling in human patients.

## Abstract (precis)

**Summary:** Analyses of *Nik^–/–^* mice and human gene expression data reveal a new pathway involved in the development of eosinophilic esophagitis in human patients and mouse models of the disease. This article has an associated First Person interview with the first author of the paper as part of the supplementary information.

## INTRODUCTION

Over the past 20 years, eosinophilic esophagitis (EoE) has emerged as a significant cause of upper gastrointestinal (GI) morbidity, with significant quality of life implications for patients (Straumann and Simon, 2005; Hruz et al., 2011; Cianferoni and Spergel, 2016; Cianferoni et al., 2015; Mukkada et al., 2017) and an estimated $1.4 billion annual healthcare cost (Jensen et al., 2015). This disorder can affect both children and adults, with symptoms that vary by age, but typically include difficulty eating, failure to thrive, dysphagia and food impaction. EoE is considered to be an allergic disease of the esophageal mucosa and is most often associated with immune responses to food such as wheat, eggs and dairy (Cianferoni and Spergel, 2016; Almansa et al., 2011; Noel et al., 2004; Sgouros et al., 2006). Consistent with these findings, T helper 2 (Th2) cells and their related cytokines are prominently involved in EoE pathogenesis (Straumann et al., 2001) and, interestingly, other atopic diseases such as allergic dermatitis or rhinitis can be comorbidities of EoE (Abonia and Rothenberg, 2012). Currently, the most successful treatment is through induction and maintenance of a strict elimination diet, although drugs such as steroids and biologics are sometimes employed, as well as physical methods such as esophageal dilation for fibrotic stenosis (Kagalwalla et al., 2006; Lucendo, 2017; Dellon and Liacouras, 2014; Cianferoni and Spergel, 2014).

EoE must be differentiated clinically and histologically from gastroesophageal reflux disease and proton-pump responsive esophageal eosinophilia, which can be difficult, given the overlap of clinical presentation, endoscopic assessment and sometimes even response to certain treatments (Dellon, 2014; Cheng, 2015; Goyal and Cheng, 2016). EoE has several characteristics that distinguish it as a separate disease entity, such as the presence of >15 eosinophils per high power field (HPF) and typical lack of response to proton pump inhibitors (Dellon, 2011), although there may well be synergy between the various diseases. EoE also differentiates itself from other more general eosinophilic GI disorders such as eosinophilic gastroenteritis by having a restricted distribution to the esophageal mucosa. The vast majority of current animal models of EoE rely on sensitization procedures to allergens or exposure to pathogens such as *Aspergillus fumigatus*, similar to protocols originally designed for pulmonary models of asthma (Mishra, 2013; Rayapudi et al., 2010). Indeed, the existing animal models used to study EoE can be problematic because this disease can present heterogeneously with a spectrum of inflammatory characteristics and additional extra-esophageal manifestations (Mudde et al., 2016). Few murine models of spontaneous esophageal eosinophilia currently exist, and those that have been described are limited to molecules that are already well studied in Th2 biology and allergy, such as IL-5, eotaxins and IL-13 (Mishra et al., 2002; Zuo et al., 2010).

Canonical NF-kappa-B (NF-κB; NFKB1) signaling is a prominent and widely studied inflammatory signaling cascade in the field of immunology. The central process of the canonical cascade is the liberation of p65 and p50 from their inhibitor, IκB, when it is post-translationally modified by the IKK group of kinases. This allows p65/p50 translocation to the nucleus and the subsequent transcription of a multitude of gene targets. In general, the canonical pathway underlies the rapid and acute cellular response to a wide variety of pathogenic and proinflammatory stimuli. However, while the canonical NF-κB signaling cascade is well characterized and regulates a large repertoire of downstream mediators, there is a relative paucity of data associated with the lesser studied noncanonical NF-κB pathway. Canonical and noncanonical NF-κB signaling are quite different in several crucial ways in terms of their individual cascades and downstream effects. For example, ligand-receptor interactions in noncanonical signaling are much more restricted compared with the canonical pathway, and are primarily composed of a specific subset of tumor necrosis factor (TNF)-related receptors (McDaniel et al., 2016). Noncanonical signaling also depends selectively on the activity of its central molecule NF-κB inducing kinase (NIK; MAP3K14) and the IKKα subunit (CHUK). In contrast to the model of simple cytoplasmic liberation of p65, as seen in canonical NF-κB signaling, the noncanonical pathway relies heavily on protein processing for preparation of its unique unit p100, encoded by the *NFKB2* gene. The IKKα subunit, activated by NIK, drives the proteolytic removal and degradation of a segment of p100, converting it to p52. Only then can the nuclear translocation of p52 and initiation of gene transcription proceed. Protein-level dynamics are not only important in positive regulation of noncanonical NF-κB; NIK is also a target of a wide array of suppressor proteins that work to destabilize and ubiquitylate it rather than affect it simply at the gene expression level. The downstream chemokine targets for gene transcription in noncanonical signaling are less defined, but appear to be both more limited in number and more specific in function. To date, four chemokines, CXCL12, CXCL13, CCL19 and CCL21, are associated with noncanonical NF-κB activation (Allen et al., 2012; Lich et al., 2007). While these chemokines can function as proinflammatory mediators and chemotactic factors for immune cell trafficking, they are typically associated with lymphoid development and maintenance. Given this targeted effect and the prominent role of mucosal-associated lymphoid tissue in the gut, it is tempting to speculate that noncanonical signaling might play an important role in maintaining immune system homeostasis at GI mucosal surfaces (McDaniel et al., 2016). Indeed, prior research from our team revealed that loss of negative regulators of noncanonical NF-κB signaling is associated with increased progression of experimental colitis and colitis-associated tumorigenesis in mice (Allen et al., 2012).

Mice having either a functional mutation (*aly/aly*) or completely lacking this essential noncanonical kinase (*Nik^−/^*^−^) have previously been described and display a variety of phenotypes, such as improper formation of secondary lymphoid organs, altered immune responses and, in *Nik*^−/−^ mice specifically, a hypereosinophilic-like syndrome (Karrer et al., 2000; Hacker et al., 2012; Yin et al., 2001; Shinkura et al., 1996). Common gross and clinical observations in these mice include dermatitis, ocular keratitis, urticaria, weight loss and microscopic deposits of eosinophils into major organs such as liver, lung and spleen. The eosinophilia in *Nik^−/^*^−^ mice seems to be dependent on T cells, as lack of lymphocytes results in prevention of the syndrome (Hacker et al., 2012). Indeed, T cells from *Nik^−/^*^−^ mice appear to be naturally more prone to a Th2 phenotype, which might be an additional driving force behind the hypereosinophilia (Hacker et al., 2012). Although these animals have previously been characterized, the presence and patterns of eosinophilic influx in the GI system, and the role of NIK and noncanonical NF-κB signaling in human eosinophilic GI disease, have not been explored.

Here, we characterized the GI tract of *Nik^−/−^* mice and determined that there is a significant and localized targeting of the esophageal mucosa by eosinophils with sparing of the rest of the GI tract. Morphologic changes such as microabscessation, basal cell hyperplasia and fibrosis all mimicked the progression of EoE in human patients. Esophageal inflammation and remodeling in these mice was accompanied by upregulation of key cytokines involved in the microenvironmental signature of EoE, along with increased transcription and protein expression of thymic stromal lymphopoietin (TSLP), which is a key player in human EoE. Metadata analysis of publically available gene expression data sets from patient and control biopsies revealed significant increases in genes associated with both the activation and repression of noncanonical NF-κB signaling in EoE-afflicted subjects. The significance of our findings go beyond identification of a spontaneous preclinical mouse model of EoE to additionally implicate a novel pathway in the development of the disease itself.

## RESULTS

### *Nik^−/−^* mice develop a spontaneous hypereosinophilic-like syndrome (characterized by ulcerative dermatitis and weight loss

**Fig. 1.**
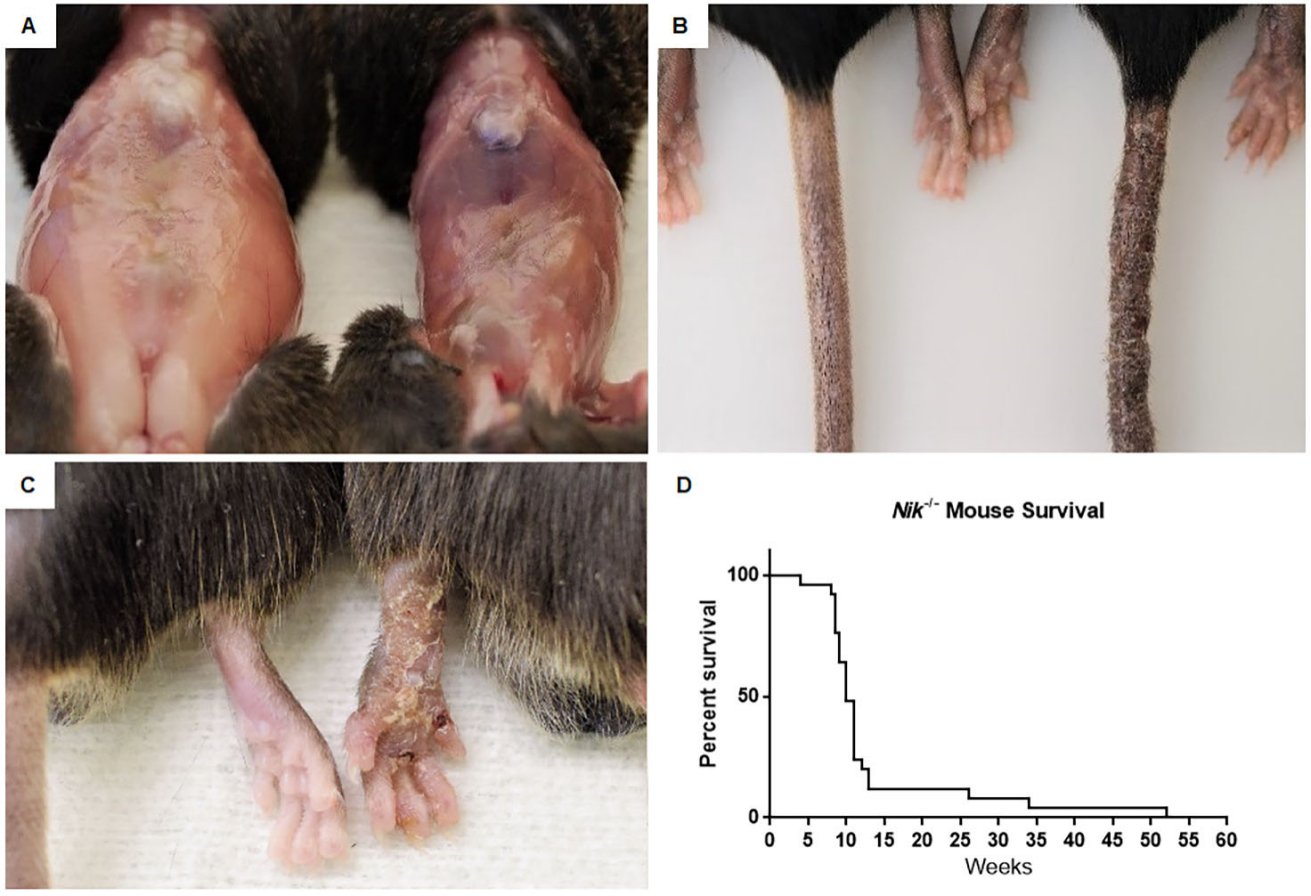
**Lack of NIK in mice results in a hyperinflammatory phenotype.** (A) *Nik*^−/−^ mice develop progressive inflammation that leads to loss of subcutaneous fat stores and overall body condition [wild-type (WT) sibling, left; *Nik^−/−^*, right]. (B,C) They also develop a scaling, ulcerative dermatitis that most commonly affects the tail (B; WT, left; *Nik^−/−^*, right) and the extremities (C; WT, left; *Nik^−/−^*, right). (D) A large proportion of *Nik^−/−^* mice require euthanasia by 12 weeks of age owing to these changes. *n*=25.

Previous studies have characterized the development of hypereosinophilic-like syndrome (HES) in *Nik^−/−^* mice (Hacker et al., 2012). However, the underlying mechanism associated with this phenotype is unclear, and a variety of local environmental factors could potentially impact disease progression. Thus, we first sought to confirm and characterize the progression of HES in the *Nik^−/−^* mice under our institutional conditions. Consistent with previous reports, we observed significant tissue eosinophilia, tissue injury, and increased morbidity and mortality. HES in these animals resulted in progressive loss of body condition (Fig. 1A) and a scaling dermatitis that most commonly affected the tail (Fig. 1B), extremities (Fig. 1C) and eventually the head, ear and shoulder region. Indeed, our animals typically require premature euthanasia at weeks 9-12, specifically due to the progressive nature of the dermatitis and associated morbidity (Fig. 1D). Onset of the dermatitis is variable, with some animals displaying lesions by as early as 3 weeks of age, and others remaining outwardly unaffected until several months of age. Both male and female animals appeared to be equally affected.

### The GI tract of *Nik^−/−^* mice displays a localized pattern of eosinophilic inflammation that targets the esophageal mucosa

**Fig. 2.**
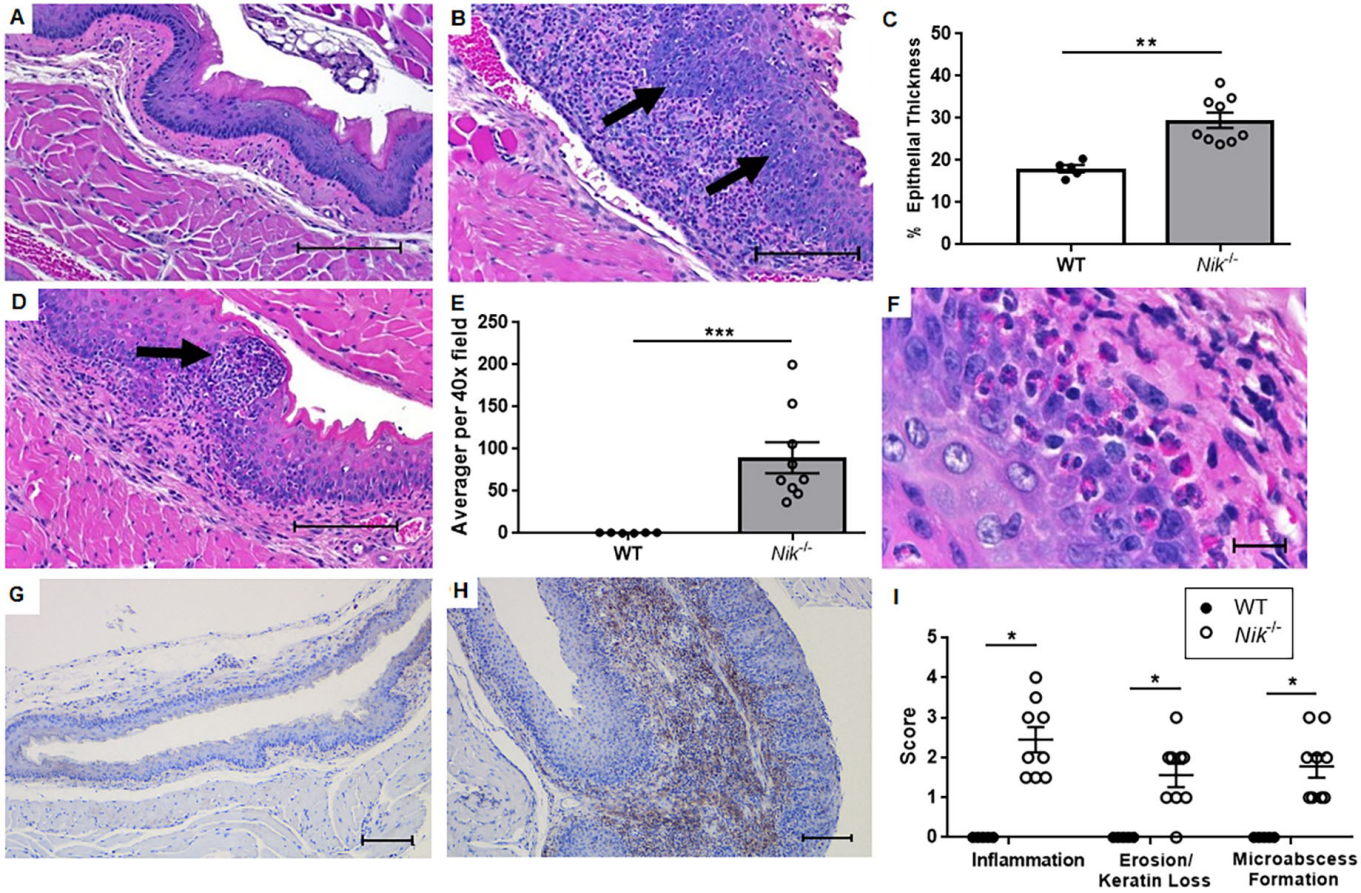
**The *Nik^−/−^* mouse esophagus is specifically targeted by eosinophils and mimics human EoE histologically.** (A-C) Compared to wild-type littermates (A), *Nik^−/−^* mice (B) display marked eosinophilic inflammation in the esophagus in both the submucosa and epithelium, accompanied by scattered lymphocytes. This change is often accompanied by basal cell hyperplasia (arrows), which was quantified as a percentage of overall epithelial thickness (C) (20× magnification; scale bar: 100 µm). (D) Eosinophils are quite prominent in the epithelial layer and occasionally form small microabscesses (arrow) (20× magnification; scale bar: 100 µm). (E) Eosinophils were enumerated at five independent points in each section and averaged, and were far above the 15-20 per HPF deemed necessary for diagnosis of EoE. (F-H) The identity of the cells as eosinophils was based on nuclear morphology (bilobed to donut shaped), correct size (9-12 µm), distinct, well-defined magenta granules (F) (100× magnification; scale bar: 10 µm), and positive reactivity to major basic protein (G,H; WT and *Nik^−/−^*, respectively) (10× magnification; scale bar: 200 µm). (I) Other features of esophagitis that were measured were severity and distribution of eosinophilic inflammation, esophageal erosion and keratin loss, and microabscess frequency, with *Nik*^−./−^ mice showing significantly increased histopathologic scores in all categories. Photomicrographs and graphs are representative of *n*=5 WT and *n*=9 *Nik*^−/−^ mice. H&E stain. Statistical analyses were performed using the Mann–Whitney U test. *P≤0.05, **P≤0.01, ***P≤0.001.

Despite the previous reports of generalized eosinophil presence in multiple filtering organs, inflammatory pathology in the GI system of the *Nik^−/−^* mouse appears to be restricted to the esophagus and the immediately adjacent gastroesophageal junction (GEJ), with the mucosa of the esophagus being particularly affected. In contrast to the wild-type littermates, the esophagus of *Nik^−/−^* mice is affected by a florid influx of eosinophils that invade the mucosa and submucosa and distort esophageal architecture (Fig. 2A,B) along with a secondary, smaller population of mature lymphocytes. This change is often accompanied by mucosal thickening (Fig. S2) and significant basal cell hyperplasia (Fig. 2B, arrows) with basal layers in some cases composing >30% of the total epithelial thickness (Fig. 2C). Eosinophils are quite prominent in the border of the squamous epithelial layer and the submucosa, and occasionally form small intraepithelial microabscesses and degranulate (Fig. 2D, arrow). These eosinophils were formally quantified and were present in densities far greater than 15 per HPF, a diagnostic criterion for the human disease (Fig. 2E). The identity of these cells as eosinophils was confirmed under high magnification, which enables the characteristic distinct magenta granules and bilobed nucleus to be appreciated (Fig. 2F), as well as immunohistochemical staining with anti-mouse major basic protein (Fig. 2G-H). Inflammation, keratin loss/erosion, and microabscessation were also histologically graded, with *Nik*^−/−^ mice showing significant increases in all of these characteristics (Fig. 2I).

### Esophageal fibrosis and GEJ hyperplasia are key sequelae to inflammation in *Nik^−/−^* mice

**Fig. 3.**
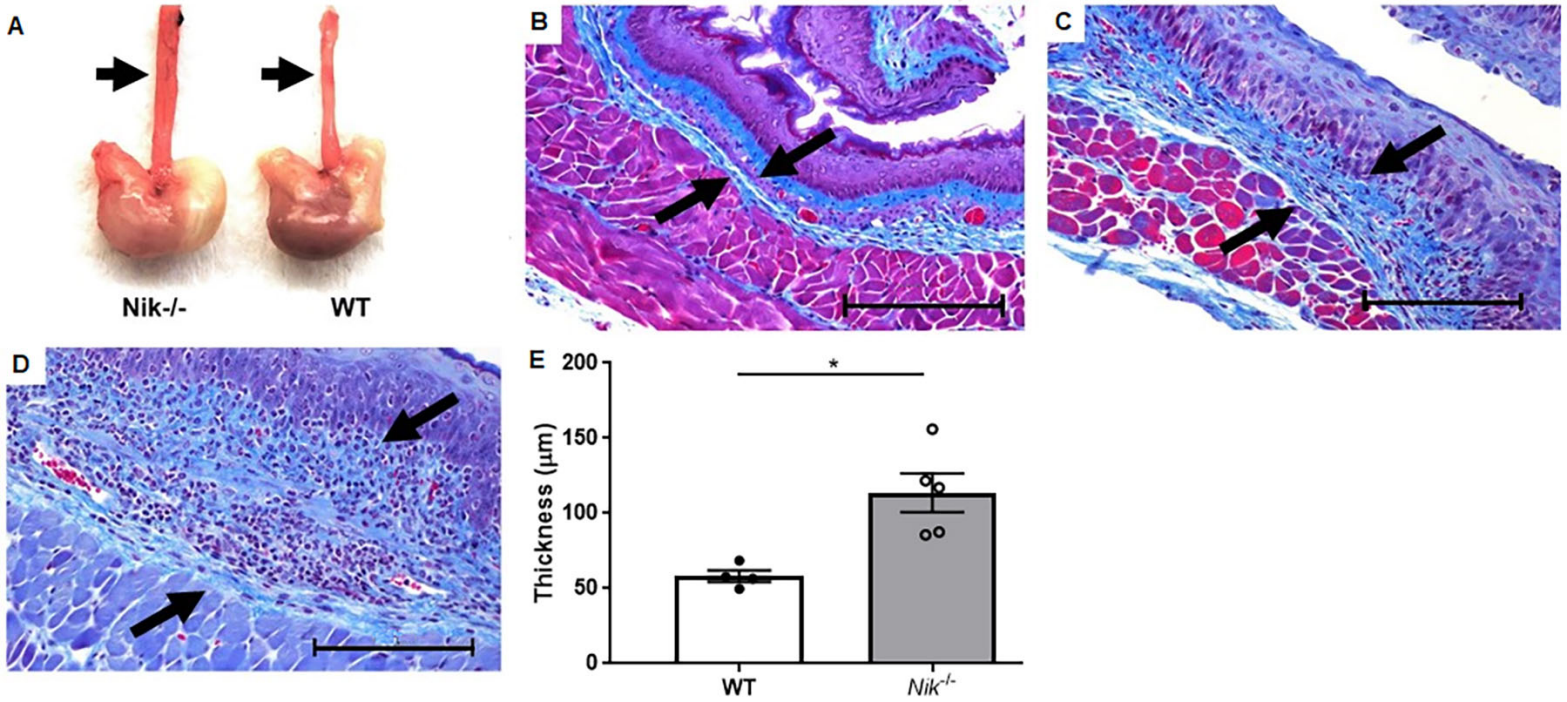
**Fibrosis is a significant sequelae to eosinophilic esophagitis in *Nik^−/−^* mice.** (A) Grossly, the esophagus of the *Nik^−/−^* mouse is circumferentially thicker than that of the WT (arrows). (B-D) Esophageal sections were stained with trichrome, revealing a consistent and significant expansion of the submucosa (arrows) by fibrous connective tissue in the *Nik^−/−^* mice (C,D) compared with WT mice (B). (E) Thickness of the submucosal collagen deposition was measured using the tunica muscularis and the basal cell layer of the epithelium as borders (arrows). Trichrome stain (20× magnification; scale bar: 100 µm). *n*=5 WT, *n*=7 *Nik^−/−^*. Statistical analyses were performed using the Mann–Whitney U test. *P≤0.05.

**Fig. 4.**
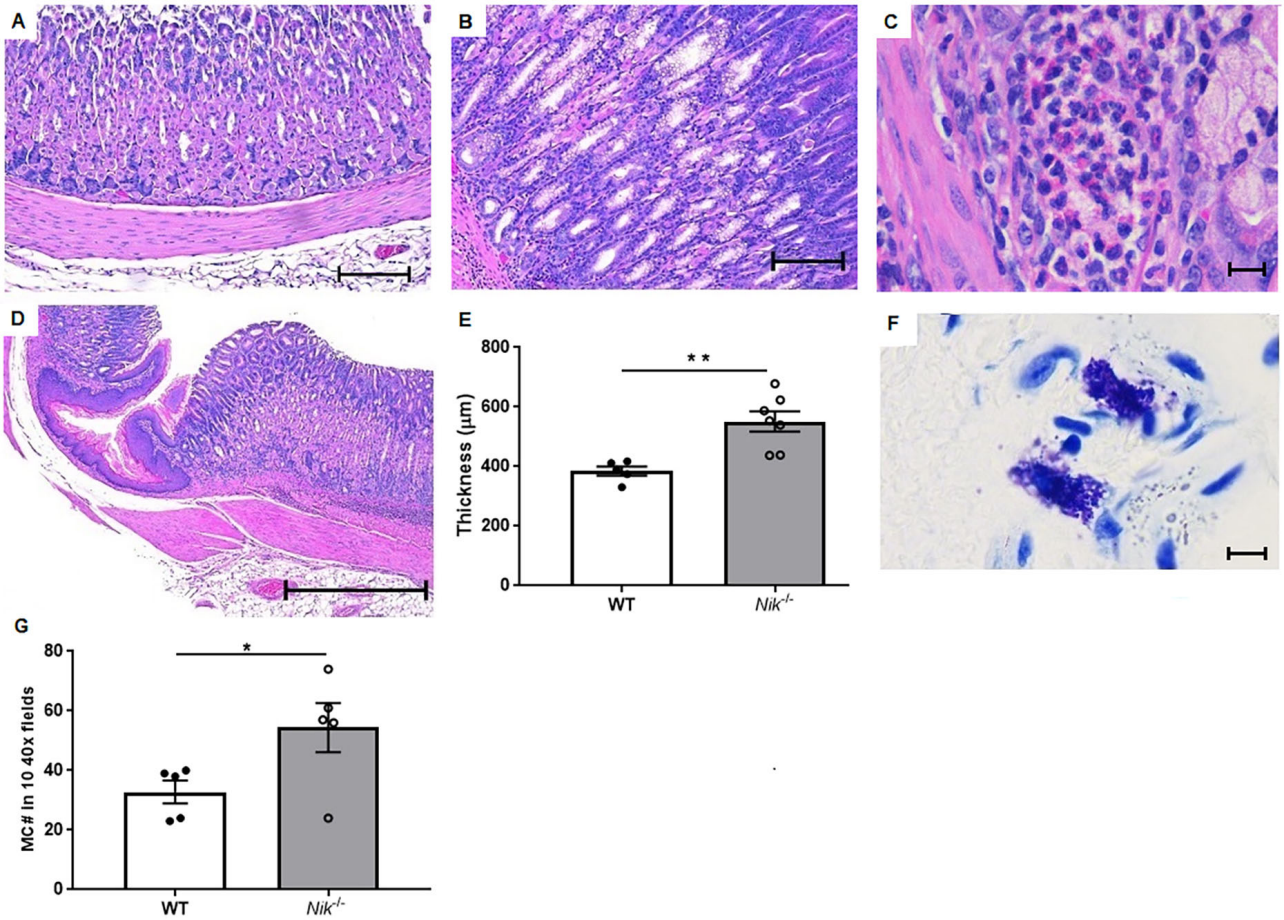
**Microscopic changes of upper GI inflammation in *Nik^−/−^* mice is limited to the esophagus and GEJ.** (A-C) Compared to wild-type littermates (A; 10× magnification; scale bar: 200 µm), *Nik^−/−^* mice (B; 10× magnification; scale bar: 200 µm) show gastric hyperplasia and localized foci of eosinophilic inflammation (C; 100× magnification; scale bar: 10 µm). (D,E) These changes are limited to the GEJ and the immediately adjacent mucosa (D; 4× magnification; scale bar: 500 µm), and the thickening was statistically significant when quantified (E). (F,G) Toluidine Blue staining showed an increase in mast cells in the GEJ area (F; 100× magnification; scale bar: 10 µm), which quantification revealed to be significant (G). For H&E staining and quantifications, *n*=5 WT, *n*=7 *Nik^−/−^*. For Toluidine Blue staining, *n*=5 WT, *n*=5 *Nik^−/−^*. Statistical analyses were performed using the Mann–Whitney U test. *P≤0.05, **P≤0.01.

Given that fibrosis and stricture of the esophagus is a serious complication of eosinophilic esophagitis in human patients (Dellon et al., 2014; Cheng et al., 2012; Nhu and Aceves, 2017), we investigated the degree of collagen deposition in the submucosa of esophageal tissue from wild-type and *Nik^−/−^* mice. Grossly, *Nik^−/−^* esophagi were thicker than those of wild-type littermates (Fig. 3A). Using trichome staining, massive expansion of the submucosa in the *Nik^−/−^* animals was apparent, with extensive collagen deposition as compared to wild-type mice (Fig. 3B-D). The degree of submucosal fibrosis was quantified using ImageJ software to measure the average submucosal diameter, with *Nik^−/−^* mice having approximately twice as much collagen deposition as wild-type mice (Fig. 3E). The mucosa of the GEJ of *Nik^−/−^* mice with eosinophilic esophagitis is also circumferentially hyperplastic and contains scattered inflammation suggestive of local irritation (Fig. 4A-D). Mucosal thickness was again quantified from the level of the apical mucosa to the basement membrane (Fig. 4E), with *Nik*^−/−^ animals showing significant thickening. While the majority of GEJ-focused inflammation was located only in that area, there was occasional mild extension into the cranial forestomach. This restricted pattern of eosinophil distribution in the GI tract is strikingly similar to that observed in human EoE patients. It is also interesting to note the concurrent dermatitis and esophagitis in the *Nik*^−/−^ mouse, as atopic dermatitis (AD) can be a co-morbidity of human EoE patients; however, whether the dermatitis of the *Nik*^−/−^ mouse is primarily an allergic phenomenon as it is in AD is unclear. Mast cells, another important inflammatory cell in EoE, were identified by Toluidine Blue staining (Fig. 4F) and counting in the esophagus and GEJ. Counts were not significantly different in the esophagus of *Nik*^−/−^ mice from those in wild-type mice (Fig. S4); however, the GEJ of *Nik*^−/−^ mice showed a significant mastocytosis compared to wild-type mice (Fig. 4G). The caudal glandular stomach, and small and large intestines were unaffected by inflammation in age-matched controls (Fig. S3), where all mucosal architecture was within normal limits. The only instance in which mild eosinophilic inflammation is seen in the lower GI tract is in older animals (>6 months), and it remains both mild and more consistent with vascular spillover from generalized hypereosinophilia rather than any sort of specific mucosal targeting, such as what is seen in the esophageal lesions.

### Gene expression profiles in the esophagus of *Nik^−/−^* mice are similar to those observed in human EoE patients, with special emphasis on TSLP

**Fig. 5.**
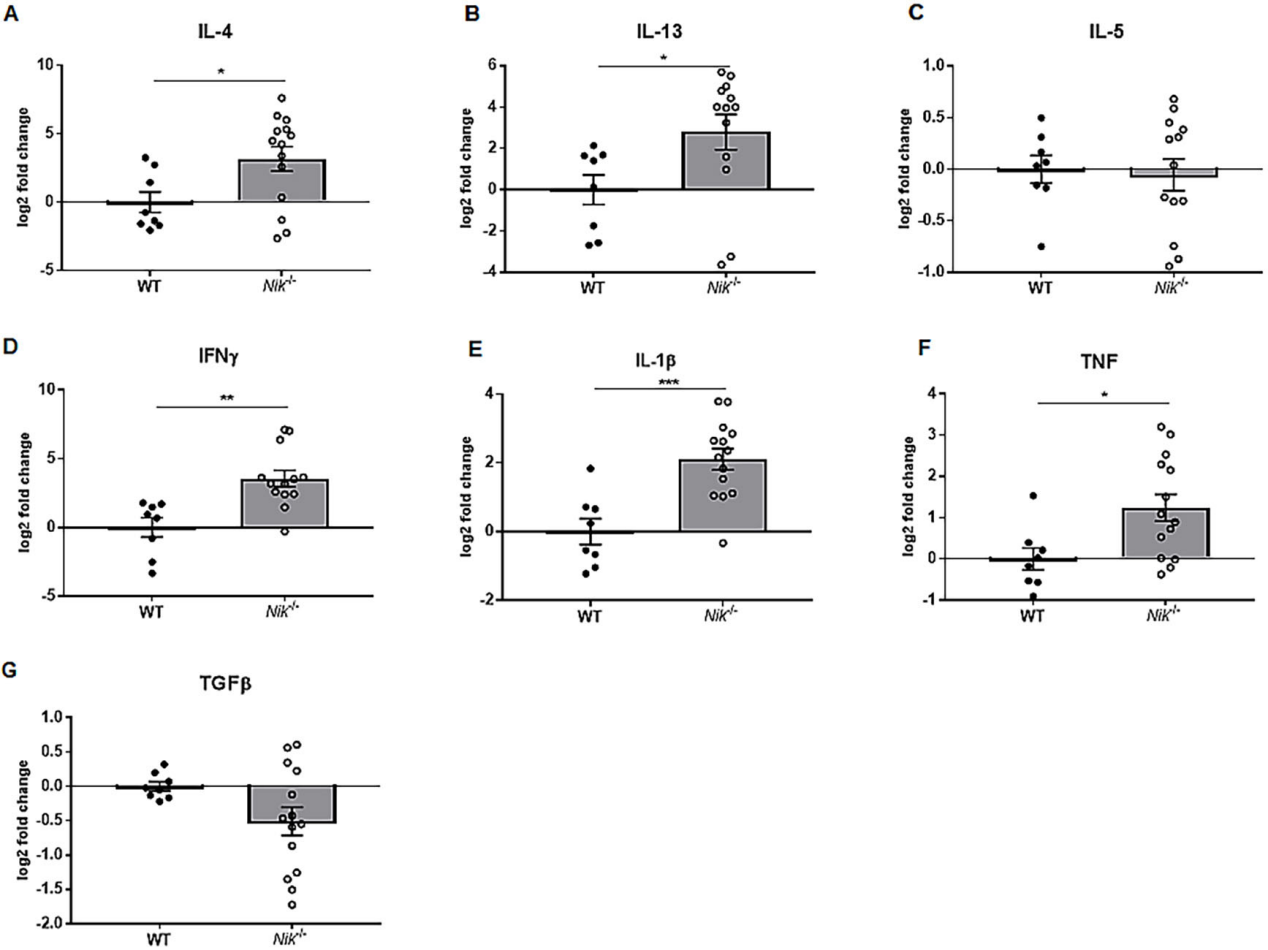
**Gene expression in the *Nik^−/−^* esophagus reveals a gene expression profile that mimics that of human EoE.** (A-C) Whole esophageal tissue was used for mRNA extraction and gene amplification. *Nik^−/−^* mice have significantly elevated expression of important Th2/EoE-relevant cytokines *Il4* (A) and *Il13* (B), with no change in *Il5* (C). (D-F) *Ifng*, *Il1b* and *Tnf* expression levels were also significantly increased. (G) Despite the fibrosis seen in the model, the expression of *Tgfb1* was not significantly different. For all genes, *n*=8 WT, *n*=14 *Nik*^−/−^. Statistical analyses were performed using the Mann–Whitney U test. *P≤0.05, **P≤0.01, ***P≤0.001.

**Fig. 6.**
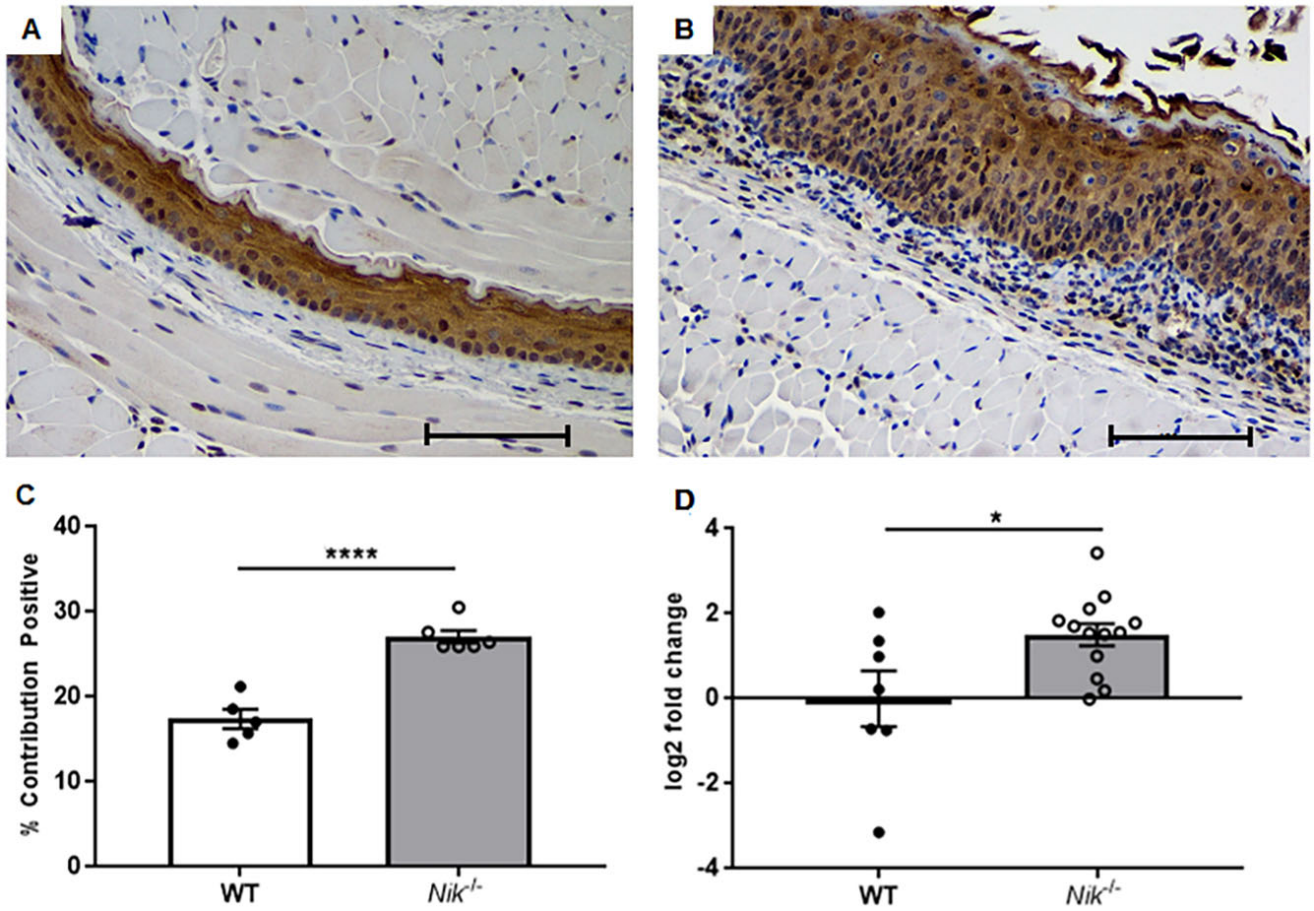
**Thymic stromal lymphopoetin plays a significant role in NIK-mediated EoE.** (A,B) Immunohistochemical staining for TSLP reveals significant expression in both WT and NIK-null epithelium (20× magnification; scale bar: 100 µm). (C) Quantification using image software revealed greater overall expression of TSLP in *Nik*^−/−^ esophagi, potentially caused, at least in part, by epithelial hyperplasia. (D) *Tslp* was also upregulated at the gene expression level in whole esophageal tissue. Histology and image quantification; *n*=5 WT, *n*=6 *Nik*^−/−^. Gene expression; *n*=8 WT, *n*=14 *Nik*^−/−^. Statistical analyses were performed using the Mann–Whitney U test. *P≤0.05, **P≤0.01, ***P≤0.001.

EoE is classically thought of as a Th2-mediated disorder characterized by increased cytokines, such as IL-4 and IL-13. Additionally, TSLP, a potent attractor and promoter of Th2 activity, has also been implicated in the pathogenesis of EoE, with some patients showing gain of function mutations (Sherrill et al., 2010). We evaluated esophageal mRNA expression of a wide panel of cytokines in our *Nik^−/−^* mice to further dissect the inflammatory microenvironment of their eosinophilic esophagitis. Esophageal tissue in *Nik*^−/−^ mice displayed significant upregulation of prominent Th2 cytokines *Il4* and *Il13* (Fig. 5A,B); however, there was no significant difference in *Il5* expression (Fig. 5C). *IL1b*, *Ifng* and *Tnf* were also upregulated (Fig. 5D-F) potentially indicating an additional Th1 contribution to disease processes as well as support for generalized inflammation and fibrosis. There was no significant difference in expression of *Tgfb1* (Fig. 5G), despite the impressive fibrosis seen in the *Nik*^−/−^ mice. TSLP was evaluated at both the gene and protein level, owing to its importance in Th2-mediated immune responses and eosinophilic esophagitis in particular. Significant elevation of *Tslp* mRNA transcripts was detected in the esophagi of *Nik*^−/−^ mice (Fig. 6C). Subsequently, immunohistochemistry was performed to confirm this finding at the protein level and evaluate the distribution and localization of TSLP within esophageal tissue. *Nik*^−/−^ mice exhibited significant TSLP immunoreactivity within the esophageal epithelium (Fig. 6A,B), that was quantitatively greater than that of wild-type mice (Fig. 6D). This overall increase in TSLP immunoreactivity appears to be caused by the hyperplastic nature of the *Nik*^−/−^ esophageal mucosa, with increased numbers of epithelial cells producing TSLP and therefore accounting for increased overall positivity of the tissue as a function of area.

### The noncanonical NF-κB signaling pathway is significantly dysregulated in human EoE patients

**Fig. 7.**
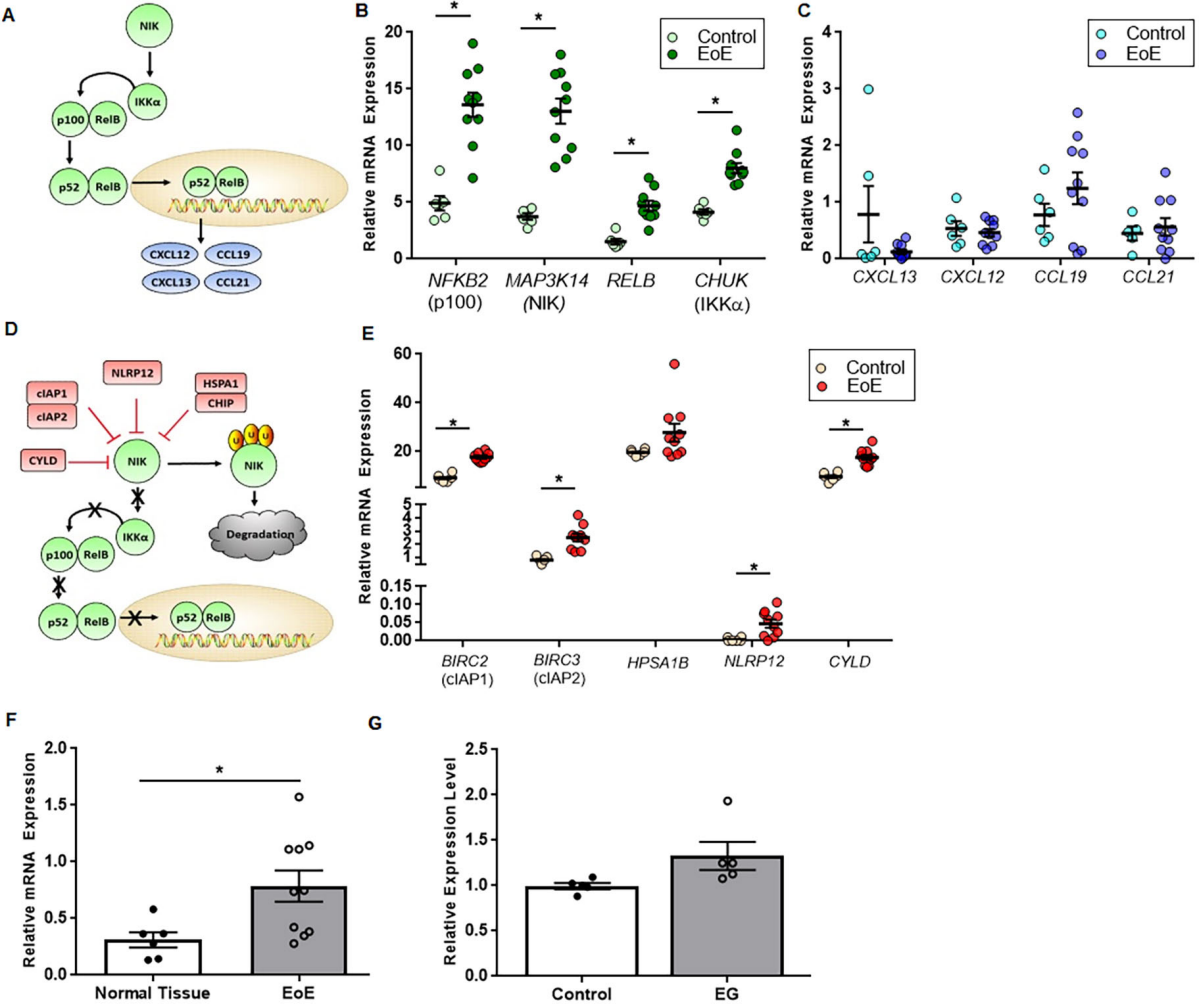
**Noncanonical gene expression patterns in human EoE patients implies blockage of NIK function.** (A) Upon ligand-receptor interaction, NIK activates IKKα, resulting in the proteolytic processing of p100 into p52 with subsequent nuclear translocation and promotion of effector chemokine gene expression. (B,C) Esophageal biopsies from human EoE patients show significantly upregulated expression of main noncanonical components *NFKB2* (p100), *NIK*, *RELB* and *CHUK* (IKKα) (B); however, there is no significant effector chemokine response, indicating a possible signaling disconnect (C). (D,E) Negative regulation of NIK takes place at the protein level via ubiquitylation and degradation (D), and all but one of molecules are simultaneously upregulated in human patients (E). (F) In addition, there is also significant upregulation of the *MAP3K14* (*NIK*) antisense RNA, which might be interfering with proper transcription. (G) There is no significant change in *MAP3K14* expression in the gastric antrum of eosinophilic gastritis patients. The EG (*n*=5 control, *n*=5 EG) and EoE (*n*=6 control, *n*=10 EoE) data sets are available from Gene Expression Omnibus under accession numbers GSE54043 and GSE58640, respectively. Statistical analyses were performed using the Mann–Whitney U test. *P≤0.05.

There is currently a paucity of data pertaining to the role of the noncanonical NF-κB signaling cascade in modulating inflammation in the GI tract. The data from our *Nik^−/−^* mice suggest that inhibition of the noncanonical NF-κB signaling pathway results in increased EoE pathogenesis. However, the role of this pathway in human patients is unexplored, and the activation state of noncanonical NF-κB signaling in EoE has yet to be evaluated. To address these shortcomings and evaluate the relevance of animal model findings in human patients, we conducted a retrospective analysis of gene expression data archived as an NIH GEO data set from human EoE patients and control patients (Sherrill et al., 2014). This study evaluated gene expression on >31,666 transcripts from six healthy controls and 10 EoE patients. Data were normalized to the average expression of five housekeeping genes and the fold change in gene expression in the EoE patients compared with the healthy controls was evaluated. Our analysis revealed a significant upregulation in specific genes associated with noncanonical NF-κB signaling (Fig. 7A) in biopsies from EoE patients compared with the healthy controls. Although several genes were significantly upregulated, defined as a greater than twofold change in expression, in the EoE patients, we found that several genes were increased >100-fold, such as *NIK* itself (629.16-fold increase), *NFKB2*/p100 (412.31-fold increase) (Table S1; Fig. 7B), and the noncanonical receptors *CD40* (180.17-fold increase) and *LTβR* (3010.012-fold increase) (Table S1). Other key components of noncanonical signaling, such as *RELB* and *CHUK* (*IKKα*), were also significantly increased (Table S1; Fig. 7B).

Together, these data would suggest a significant increase in the transcription of downstream chemokines *CXCL12*, *CXCL13*, *CCL19* and *CCL21*. Likewise, these observations run counter to our findings in the *Nik^−/−^* mice, in which reduced noncanonical NF-κB signaling is correlated with increased disease progression. However, we did not observe a significant difference in genes directly downstream of noncanonical NF-κB pathway activation in the human EoE patients (Fig. 7C). These findings can be reconciled by the corresponding findings that powerful negative regulators of the noncanonical pathway (Fig. 7D) were also significantly upregulated in the patient specimens (Fig. 7E). Specifically, our data revealed significantly increased transcription of the genes encoding cIAP1/2 (BIRC2/3), CYLD, NLRP12, HSP1 and CHIP (STUB1) in human EoE patients (Fig. 7E). These regulators are known to potently inhibit multiple levels of the noncanonical NF-κB signaling cascade, including functioning either directly or indirectly as post-translational degraders and destabilizers of NIK (reviewed in McDaniel et al., 2016). Thus, it is possible that despite upregulation at the gene level, blockade and subsequent degradation of NIK at the protein level is able to outpace its transcription. This scenario would agree with the loss of functional NIK in our knockout mice and the similar EoE phenotype between the animal model and human patients. Also of note, we also found a significant upregulation in *MAP3K14* (*NIK*) antisense RNA in the biopsy samples from the EoE patients, which could be interfering with translation and further repressing this pathway (Fig. 7F). Lastly, we analyzed *NIK* expression in the gastric antrum of eosinophilic gastritis patients and found that there was no significant difference between patient populations (Fig. 7G), highlighting the potential specificity of the esophageal mucosa in this disease process. Together, these data reveal the presence of a complex, dynamic regulatory environment associated with noncanonical NF-κB signaling during EoE, and identify a large number of previously undefined mediators that appear to modulate disease progression in human patients.

## DISCUSSION

The noncanonical NF-κB pathway remains an understudied contributor to GI disease in general and particularly in its modulation of eosinophilic inflammation. Here, we not only present the *Nik^−/−^* mouse as a potential spontaneous model of eosinophil influx during EoE, but also identify dysregulation of the noncanonical NF-κB signaling cascade as a potentially significant contributor in disease pathogenesis. The similarity in microscopic features and severity in this spontaneous model is on par with the disease classically induced by *Aspergillus* and other allergen/agent-induced models of EoE (Mishra et al., 2001; Rayapudi et al., 2010; Niranjan et al., 2013). However, although the *Nik^−/−^* mice are excellent models of specific aspects of disease progression, there are certainly limitations as there are with all rodent models. For example, the *Nik^−/−^* mice have increased circulating eosinophils (Hacker et al., 2012), whereas human EoE patients typically do not present with high levels of systemic eosinophilia. Indeed, the ultimate progression of the *Nik^−/−^* mice to systemic HES reflects broader mechanistic defects beyond the esophagus and is a limitation of this proposed model. However, the *Nik^−/−^* mouse remains one of the few spontaneous models of esophageal esophagitis, and demonstrates significant microscopic and gene expression patterns similar to the human disease. The eosinophilic targeting of the esophagus and the concurrent sparing of the caudal GI system of the *Nik*^−/−^ mouse is intriguing given the categorization of HES as a systemic disease. It is possible that, given the fact that severe dermatitis is most often the clinical sign that leads to euthanasia, that lack of noncanonical NF-κB signaling is essential for homeostasis in areas containing stratified squamous epithelium, such as the esophagus and skin in the mouse. This would be consistent with prior studies that revealed local hypersensitivity in transgenic mice that overexpress IL-5 in squamous epithelial cell compartments (Masterson et al., 2014). Because esophageal epithelial cells can act as nonprofessional APCs in EoE (Mulder et al., 2011), lack of NIK could well be key in the epithelial-driven promotion of a Th2 microenvironment and the concurrent accumulation of eosinophils. Alternatively, lack of noncanonical signaling in either eosinophils or T lymphocytes might result in excessive targeting of squamous structures, with inflammation of other organ systems being a product of vascular spillover from peripheral hypereosinophilia. Given that the HES is abrogated when *Nik*^−/−^ mice are backcrossed onto immunodeficient mice lacking T and B cells, it appears that the implications of NIK loss in the adaptive immune system is the primary issue (Hacker et al., 2012). As noncanonical NF-κB signaling is intricately involved in lymphoid development and structure, it is tempting to speculate that dysregulated T cell biology is a major player in NIK-mediated EoE development. This concept is supported by recent studies focused on CD40L (CD40LG), a ligand in the noncanonical NF-κB signaling cascade that is expressed on T cells. These recent studies revealed a strong correlation between CD40L and EoE pathogenesis (Blanchard et al., 2011). Indeed, CD40L expression, along with the expression of an array of additional, well-known proinflammatory cytokines, such as IL-4, IL-5, IL-13 and IL-17, were found to be highly efficient biomarkers of EoE pathogenesis (Blanchard et al., 2011). A baseline structural or junctional defect in *Nik*^−/−^ squamous epithelial cells which attracts eosinophilic inflammation as a damage control mechanism, could also be a contributor to this process and remains to be investigated. Barrier defects in the esophagus have also been seen in human EoE (Simon et al., 2015, 2017). However, the lack of significant inflammation (compared with the esophagus and skin) in the squamous epithelium of the *Nik*^−/−^ mouse forestomach distal to the GEJ junction implies that there may be other factors at play besides simple epithelial type. Finally, EoE develops as a response to a dietary allergen or allergens, which on the surface is not a feature of our model. However, the potential sensitivity of *Nik*^−/−^ mice to allergens commonly found in standard rodent chow such as wheat and corn (allergens that are also human EoE triggers) has not been investigated. Given the Th2-prone, hyperinflammatory profile of the *Nik*^−/−^ mouse, predisposition to allergy might well be a feature. Future studies using diet alteration and oral allergen exposure in these mice would be interesting to pursue. Mast cell numbers were not increased in the esophagus of *Nik*^−/−^ mice, although they were indeed elevated in the area of the GEJ – another slight deviation from the human condition.

In terms of gene expression, the upregulation of inflammatory cytokines, such as IL-4 and IL-13, that we observed in the *Nik^−/−^* mice correlated well overall with expression patterns in human EoE, with the exception of a lack of significant differences in IL-5 and TGFβ. Our characterization of TSLP in these mice was particularly rewarding given the importance of TSLP in both EoE and eosinophilic diseases as a whole. TSLP is primarily produced by nonhematopoietic cells such as fibroblasts and epithelial cells, although there is also evidence of mast cell, dendritic cell and airway smooth muscle cell expression (Takai, 2012). Upon introduction of an allergen, TSLP stimulates epithelial and stromal cells to produce T-cell-attracting substances and promote neighboring dendritic cell maturation and activity. In EoE patients, TSLP expression is generally localized to the suprabasal layer of the esophageal epithelium and can be overexpressed or mutated in EoE patients (Chandramouleeswaran et al., 2016; Noti et al., 2013; Sherrill et al., 2010; Rothenberg et al., 2010; Kottyan et al., 2014). Indeed, cultures of differentiated esophageal epithelial cells alone can produce TSLP in reaction to food allergens, such as ovalbumin *in vitro* (Chandramouleeswaran et al., 2016). In terms of other tissues composed of stratified squamous epithelium, such as skin, expression of TSLP has been shown to contribute to disease in allergic dermatitis in both human patients and mouse models (Soumelis et al., 2002; Kim et al., 2013). In our mice, TSLP was strongly expressed in all layers of the epithelium in both wild-type and Nik-null animals. Given the epithelial hyperplasia seen in the knockout animals, it appears that increased numbers of TSLP-expressing epithelial cells is the reason for the upregulation seen at the gene level rather than an increase in intensity per epithelial cell as seen by the similar staining density. Additionally, there appear to be small numbers of stromal cells, most likely fibroblasts or dendritic cells, within the submucosa of inflamed *Nik^−/−^* animals that could also be contributing to the increased expression.

While we have shown a strong Th2 phenotype in our mice that corresponds directly with human EoE, we also saw changes in other cytokines that are worthy of note, such as Th1 mediators IL-1β, IFNγ and TNF, all of which can also contribute to inflammation and EoE pathogenesis. TNF is upregulated in EoE patients and tissue (Blanchard et al., 2006; Straumann et al., 2001), and is thought to play a role in fibrotic change along with IL-1β (Aceves et al., 2007; Muir et al., 2013), although these markers are not specific for EoE (Blanchard et al., 2011). Similarly, IFNγ has been reported to be upregulated in EoE patients (Gupta et al., 2006) and was increased in more modern transcriptomic studies as well (Sherrill et al., 2014). Interestingly, CD8+ T cells from EoE patients also produce increased levels of this cytokine along with TNF (Sayej et al., 2016). Despite the significant fibrosis associated with this disease, there was not a significant upregulation in TGFβ, a very powerful and well-studied cytokine in esophageal remodeling and fibrosis in EoE (Nhu and Aceves, 2017; Cheng et al., 2012; Rieder et al., 2014), in our mice. However, even in classical EoE patients, TGFβ is not always upregulated (Lucendo et al., 2011; Straumann et al., 2010). It is possible that fibrosis in the inflamed esophagus of *Nik*^−/−^ mice might occur through a TGFβ-independent mechanism, such as fibroblast growth factor 9 (FGF9), which is elevated in EoE patients (Mulder et al., 2009; Lucendo et al., 2011). It is also possible that the fibrosis in our animals could be a primary defect associated with NIK loss rather than a cytokine-driven reaction to eosinophilic inflammation.

The upregulation of several key components of noncanonical NF-κB signaling in human EoE patients including *NFKB2* (which encodes the p100 subunit), *RELB* (a p52 chaperone), *NIK* itself, and the essential kinase IKKα (encoded by *CHUK*) in human EoE biopsy samples initially appeared contrary to our findings in our *Nik*^−/−^ mice. This was further confounded by the lack of upregulation of noncanonical effector chemokines, which one would think to be quite upregulated given the increased expression of their promoters. One of the distinguishing features of noncanonical NF-κB signaling is its extensive reliance on post-translational events, including protein processing, stabilization and ubiquitylation, that are not always correlated or reflected at mRNA levels. For example, NIK is regulated at the protein level by several other molecules, such as cIAP1/2 and NLRP12, and this stabilization (rather than expression) is key for downstream p100-p52 processing and subsequent nuclear translocation (Allen et al., 2012; Zarnegar et al., 2008; Qing et al., 2005). Interestingly, in the EoE patient data, we found a significant increase in gene expression for a diverse range of these negative regulators of noncanonical NF-κB signaling, especially those that target NIK. The most notable are cIAP1 and cIAP2, which bind to NIK to form a ubiquitin ligase complex along with TRAF proteins that eventually undergo proteosomal degradation (Zarnegar et al., 2008; Vallabhapurapu et al., 2008). This degradation is potent enough that even in the face of active TNF stimulation, noncanonical NF-κB activity is suppressed (Gentle et al., 2011). Similar to increased *cIAP1/cIAP2*, we also found significant upregulation of *CYLD*, *HSPA1B* and *FN14*, which also exert significant negative pressure on noncanonical NF-κB signaling either directly or indirectly via targeting NIK. Given this large array of post-translational destabilizers that are upregulated, it is reasonable to suggest that the gene overexpression we see in *NIK* is a compensatory reaction to overactive and continual degradation of the NIK protein. This is in line with the lack of response we see in chemokine production associated with the noncanonical NF-κB cascade and would be consistent with the findings from our *Nik*^−/−^ mice. Of course, analysis of NIK stability and additional studies of these negative regulators at the protein level in human specimens would be needed to confirm this potential mechanism, requiring the analysis of lysates from human patient biopsy samples. Another possibility for the discrepancy may be the method of tissue acquisition. Biopsy samples from human patients tend to be composed of epithelial layers only, while our sections involved the entire esophagus including submucosa and tunica muscularis, and could therefore include changes exerted by stromal cells as opposed to a purely epithelial signature.

In conclusion, we have identified a novel signaling pathway in EoE that is dysregulated in human patients and associated with spontaneous development of eosinophilic esophageal inflammation in mice. We anticipate that future studies will better define the role of NIK and noncanonical NF-κB in EoE. Here, we have uncovered an extensive repertoire of genes that have not previously been associated with EoE that might serve as future therapeutic targets or biomarkers of disease progression. Likewise, the *Nik^−/−^* mice will be highly beneficial to study eosinophil, lymphocyte and epithelial cell/stromal interactions associated with disease pathobiology. Noncanonical NF-κB signaling remains an undercharacterized pathway in mucosal biology, particularly in GI disorders. However, it is becoming more apparent that this pathway underlies a variety of mechanisms associated with aberrant inflammation in the gut and deserves greater scrutiny.

## MATERIALS AND METHODS

### Mouse models

*Nik^−/−^* mice [Map3k14^tm1^Rd^s^; originally created by Yin et al. (2001)] were generously provided by Amgen. Heterozygous mice were bred and littermates genotyped upon weaning (Fig. S1). Knockout animals and wild-type littermates were then separated by sex and genotype, and all mice were housed under specific pathogen-free conditions. Mice were fed a standard Tekland rodent diet (16% protein with ingredients in order of inclusion: wheat, corn, wheat middlings, soybean meal, corn gluten meal, soy oil) and given water *ad libitum*. Enrichment was provided in the form of red plastic huts, paper twists and Nestlets. All studies were conducted in accordance with the Institutional Animal Care and Use Committee guidelines of Virginia Maryland College of Veterinary Medicine and National Institutes of Health Guide for the Care and Use of Laboratory Animals. Mice were sacrificed at 3-20 weeks of age, depending on appearance of dermatitis, the most consistent and recognizable sign of disease onset. Age-matched wild-type animals were sacrificed at identical time points.

### Tissue collection and histological analysis

Mice were euthanized by carbon dioxide narcosis followed by cervical dislocation. The entire esophagus from the level of the GEJ to the pharynx was dissected out, flushed, opened and ‘Swiss rolled’ for histopathology evaluation. The intestine and colon were similarly prepared. Samples were fixed in 10% buffered formalin, paraffin embedded, sectioned at 5 μm and stained with hematoxylin and eosin (H&E). Esophagi were scored using designations of severity of eosinophilic inflammation (0, absent; 1, scattered eosinophils within the epithelium with or without occasional submucosal extension; 2, moderate and consistent presence of eosinophils in both the epithelium and submucosa with disruption of normal architecture; 3, severe, dense eosinophilic infiltrate with significant obscuring of the basal layer and submucosal expansion; and 4, very severe, dense infiltrate with transmural extension), keratin loss (0, absent; 1, <25% of section; 2, 26-50% of section; 3, >50% of section), and microabscessation (0, none; 1, 1-2 per esophageal roll cross-section; 2, ≥3 per esophageal cross-section with degranulation). Eosinophil count was performed based on eosinophil morphology (correct size, bilobed nucleus, prominent and distinct magenta granules), as well as positive major basic protein staining in the esophagus, small intestine (ileum) and colon (entire length). For the esophagus, five randomly selected fields were counted and averaged; for the lower GI tract, the total number in ten 40× fields was reported. Mucosa thickness in the stomach and esophagus was quantified using five randomly selected areas of mucosa surrounding the junction of each individual specimen. Basal cell hyperplasia was evaluated as a percentage of total epithelial thickness measured at five independent points within each individual sample. To evaluate collagen deposition, additional slides were stained with Masson's trichrome. Thickness of the submucosal collagen deposition was measured using the tunica muscularis and the basal cell layer of the epithelium as borders, and was quantified averaging five measurements at randomly determined points within each individual section. Toluidine Blue staining was performed using a 1% solution of Toluidine Blue O (Sigma-Aldrich) dissolved in 70% ethanol and adjusted to pH 2.3. All histopathology assessments were conducted by a board-certified veterinary pathologist (K.E.) who was blinded to the identity of the samples.

### Immunohistochemistry

Tissue sections (5 μM) were deparaffinized, hydrated and subjected to heat-mediated enzymatic retrieval using pH 6.0 citrate buffer for anti-TSLP protein staining. For anti-major basic protein staining, enzymatic antigen retrieval was performed in 0.1% trypsin for 20 min at 37°C. Endogenous peroxidase activity blocking, general serum blocking, and washings were performed using reagents from the Pierce Peroxidase IHC Detection Kit (Thermo Fisher Scientific) according to the manufacturer's protocol. Sections were incubated with rabbit anti-mouse major basic protein antibody (My BioSource, MBS2004321, 1:1000) or rabbit anti-mouse/human TSLP (Cell Signaling Technology, PA5-20320, 1:1000) at 4°C overnight. Sections were then incubated with SignalStain^®^ Boost IHC Detection Reagent (HRP, Rabbit; Cell Signaling Technology, #8114) for 30 min at room temperature and staining was detected using the Pierce Peroxidase Substrate solution and 3,3′-diaminobenzidine (DAB) following the manufacturer's protocol. The sections were counterstained with Mayer's Hematoxylin (Sigma-Aldrich), dehydrated, and mounted. Degree of immunoreactivity was measured as a percentage contribution of positive pixels per image using identical magnifications and orientations of esophageal tissue using the IHC Profiler plugin for ImageJ, and five independent measurements were taken and averaged for each individual sample.

### Gene expression

Esophageal tissue was finely minced, total RNA extracted using Trizol (Thermo Fisher Scientific), and quantity/quality assessed via Nanodrop. Complimentary DNA was made using an ABI High Capacity cDNA kit, in accordance with the manufacturer's protocols and 1 μg amplified using the Taqman-based rtPCR platform (Thermo Fisher Scientific) on an ABI 7550 Fast Block Thermocycler. Gene expression was determined using the ΔΔCt method (Livak and Schmittgen, 2001). All data were normalized to *Gapdh* and the fold change in gene expression was determined for *Tslp*, *Il4*, *Il13*, *Il5*, *Ifng*, *Tnf*, *Tgfb1* and *Il1b*. All samples were run in triplicate.

### Human gene expression analysis

Gene expression from human patient and control biopsies was evaluated using publically accessible data sets (NIH GEO) as previously described (Rothschild et al., 2017; Coutermarsh-Ott et al., 2016). Data sets were analyzed using Ingenuity Pathways Analysis (IPA) software to identify genes and pathways that were significantly dysregulated in EoE patients and specimens. Specific genes of interest identified in the IPA analysis associated with noncanonical NF-κB signaling were further evaluated. Gene expression data were normalized to the average expression of six housekeeping genes (*18S*, *ACTB*, *RPLP0*, *HPRT*, *B2M* and *GAPDH*) for each specimen, and the fold change in gene expression between EoE patient and control specimens was determined. Gene expression analysis was conducted using the array data series GSE58640 and GSE8853.

### Statistical analysis

Data were analyzed using GraphPad Prism, version 7 (GraphPad Software, Inc., San Diego, CA). Student's two-tailed *t*-test was used for comparison of two experimental groups. Multiple comparisons were performed using one-way and two-way ANOVA where appropriate followed by Mann–Whitney or Tukey post-test for multiple pairwise examinations. Correlation was also computed using GraphPad Prism. Changes were identified as statistically significant if *P*≤0.05. Data are presented as mean±s.e.m.

## Supplementary Material

Supplementary information

First Person interview
