# Supplementary material for: Noncanonical NF-κB signaling and the essential kinase NIK modulate crucial features associated with eosinophilic esophagitis pathogenesis
Source: Dis Model Mech. 2017 Dec 1;10(12):1517–27. doi: 10.1242/dmm.030767 (PMC5769607; doi:10.1242/dmm.030767)
Supplement: Supplementary information [file dmm-10-030767-s1.pdf]

Table 1: Significant fold changes in molecules directly and peripherally related to noncanonical signaling in EoE patients

Gene	Fold Change	Gene	Fold Change
CD40	180.17	***NIK***	629.16
TWEAK	2.92	***CIAP1***	288.48
TNFRSF1A	378.05	***CIAP2***	3.32
TNFRSF1B	2.05	***A20***	3.02
LTBR	3010.012	***IKKγ***	8.7
TRAF2	2.72	***IKKα***	14.56
TRAF3	2.64	***IKKβ***	91.31
FN14	5.3	***NFKB2***	412.31
RELB	8.84		

Disease Models & Mechanisms • Supplementary information

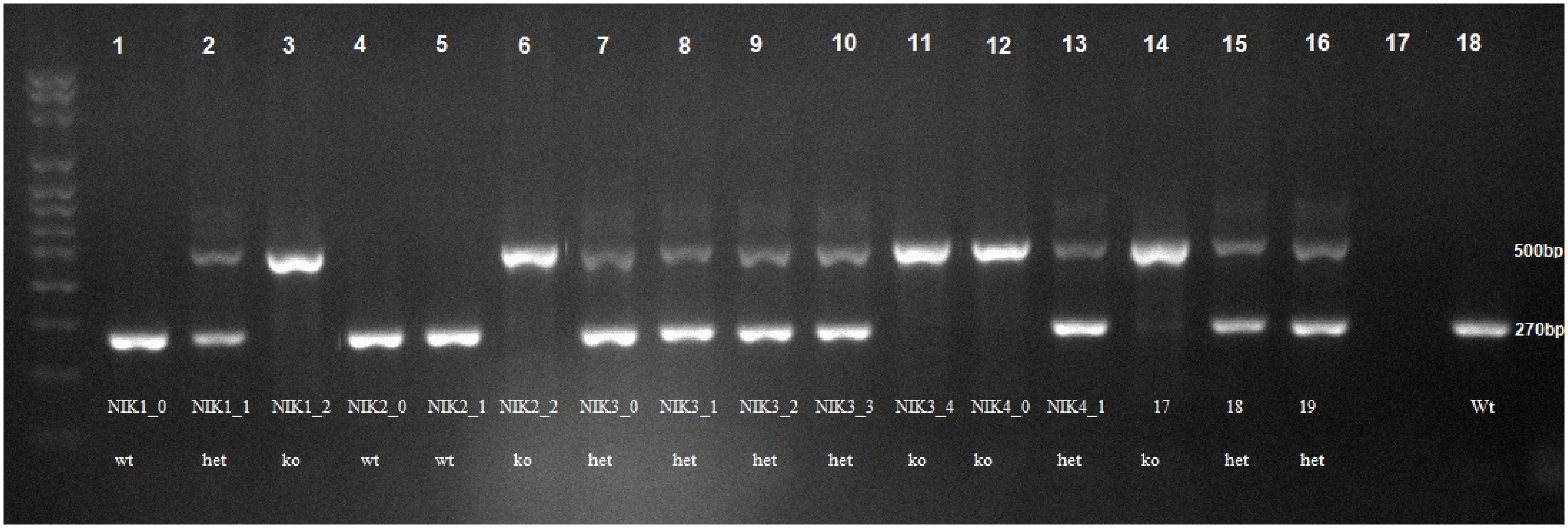

Supplementary Figure 1: ***Nik***$^{-/-}$ **mice were bred heterozygously for the generation of wild-type and knockout littermates and routinely genotyped.** An example 1.5% agarose gel showing several groups of mice (**Lanes 1-16**) with appropriate no template controls (**Lane 17**) and control/known wild-type DNA (**Lane 18**). "Wt" signifies *Nik*$^{+/+}$, "het" signifies *Nik*$^{+/-}$, and "ko" signifies *Nik*$^{-/-}$. Wild-type band = 270bp, knockout = 500bp.

Disease Models & Mechanisms • Supplementary information

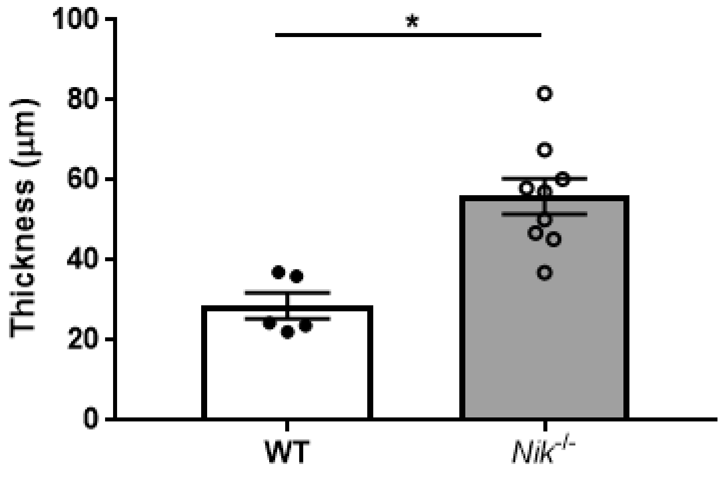

Supplementary Figure 2: Mucosal proliferation is a feature of EoE in *Nik*$^{-/-}$ mice

Nik$^{-/-}$ mice exhibit and overall thickening of the esophageal mucosa. N = 5 WT, 9 *Nik*$^{-/-}$.

Statistics were performed using the Mann-Whitney U test and significance set at p = 0.05.

Disease Models & Mechanisms • Supplementary information

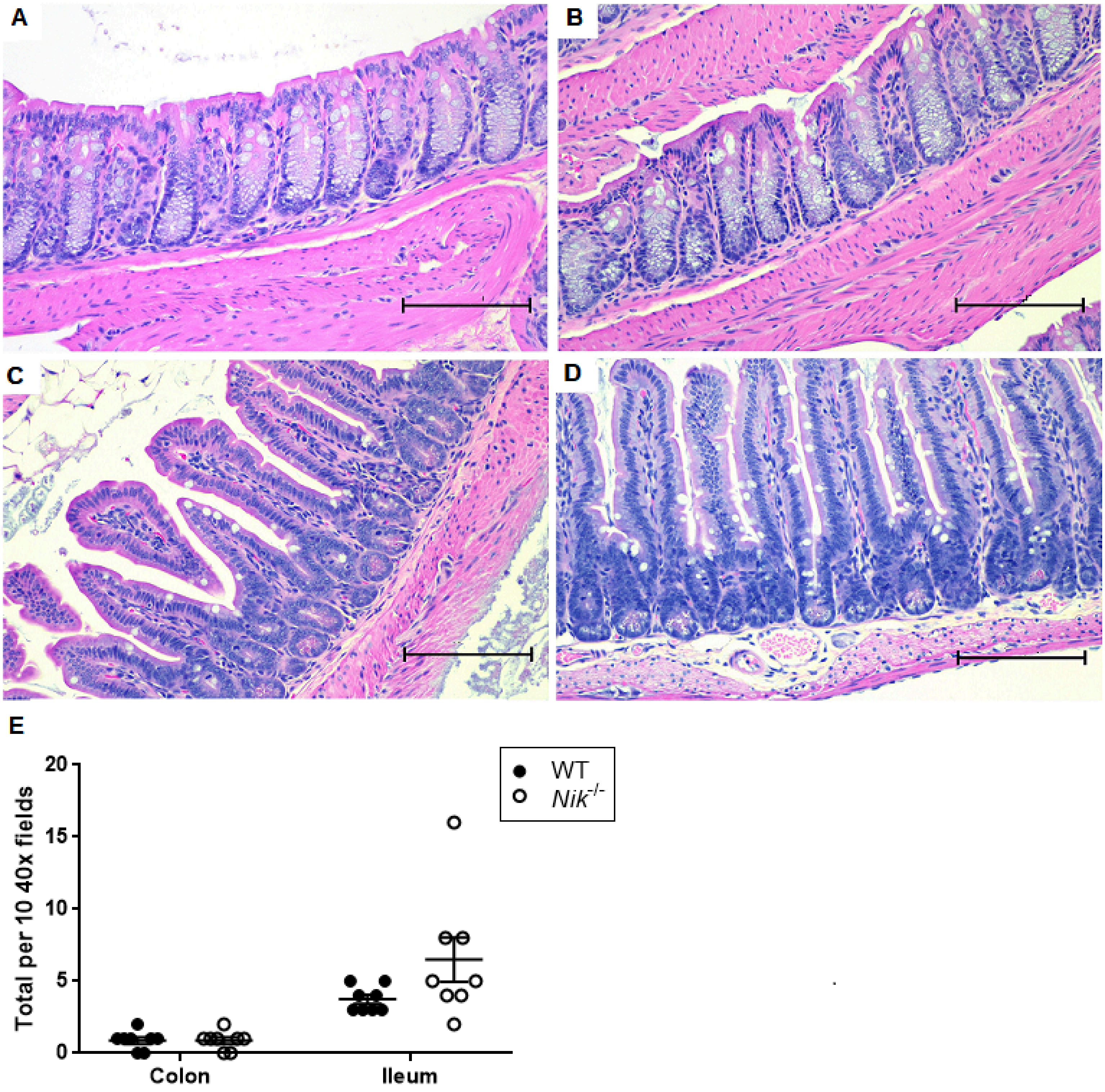

Supplementary Figure 3: Eosinophilic inflammation in the GI tract is localized to the esophagus in *Nik*$^{-/-}$ mice. (A-D) Compared to **(A)** wild-type colon (20X bar = 100µm) and **(C)** small intestine (20X, bar = 100mm), the lower gastrointestinal tract of *Nik*$^{-/-}$ mice including the **(B)** colon (20X) and **(D)** small intestine (20X) was within normal limits. **(I)** Eosinophil counts (total number per 10 40X fields, n = 8 per group) in the lower GI were not significantly different between WT and *Nik*$^{-/-}$ and there was no mucosal architecture disruption. N = 8 WT, 8 *Nik*$^{-/-}$. H&E stain. Statistics were performed using the Mann-Whitney U test and significance set at p = 0.05.

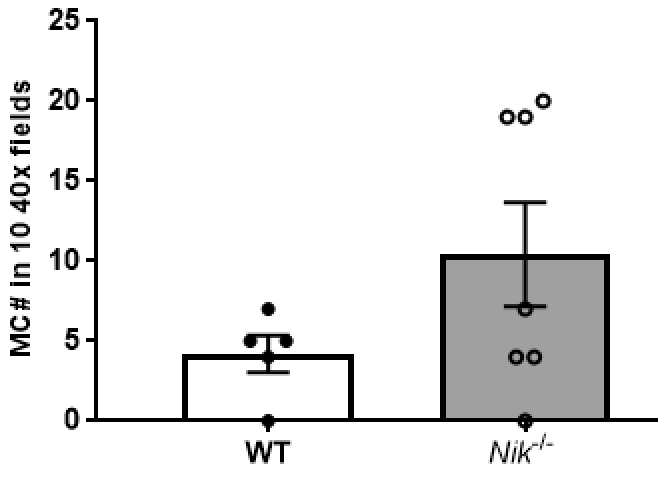

Supplementary Figure 4: Mast cell densities in the inflamed esophagi of *Nik*$^{-/-}$ mice are not significantly different that wild-type mice. Esophageal mast cell counts based on toluidine blue staining were not significantly different between WT and *Nik*$^{-/-}$ mice. N = 5 WT, 7 *Nik*$^{-/-}$. Mast cell counts are expressed as total number of mast cells in 5 40x fields for each individual sample. Statistics were performed using the Mann-Whitney U test and significance set at p = 0.05.

Disease Models & Mechanisms • Supplementary information
