# Supplementary material for: Noncanonical NF-κB signaling and the essential kinase NIK modulate crucial features associated with eosinophilic esophagitis pathogenesis
Source: Dis Model Mech. 2017 Dec 1;10(12):1517–27. doi: 10.1242/dmm.030767 (PMC5769607; doi:10.1242/dmm.030767)
Supplement: First Person interview [file dmm-10-030767-s2.pdf]

© 2017. Published by The Company of Biologists Ltd | Disease Models & Mechanisms (2017): doi:10.1242/dmm.030767: Supplementary information

FIRST PERSON

First person – Kristin Eden

First Person is a series of interviews with the first authors of a selection of papers published in Disease Models & Mechanisms, helping early-career researchers promote themselves alongside their papers. Kristin Eden is first author on 'Noncanonical NF-κB signaling and the essential kinase NIK modulate crucial features associated with eosinophilic esophagitis pathogenesis', published in DMM. Kristin is a PhD student in the lab of Irving Coy Allen at Virginia Maryland College of Veterinary Medicine, Virginia, USA, investigating the relationship between inflammation and cancer in the gastrointestinal tract.

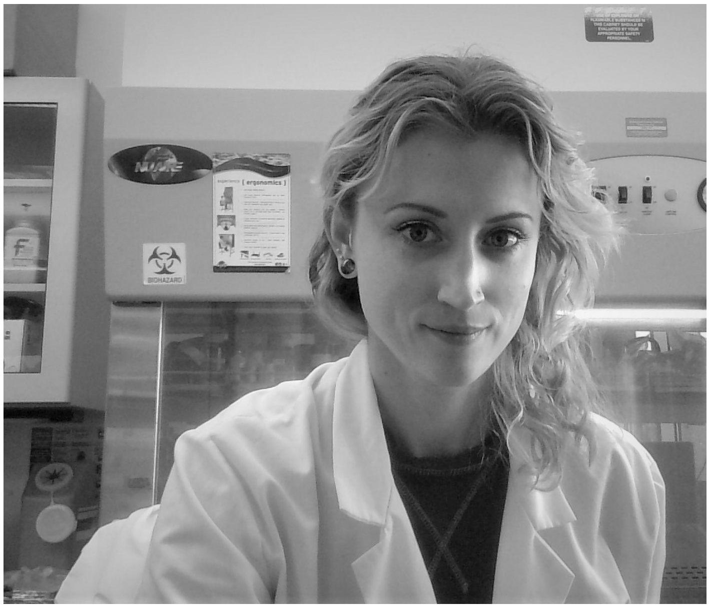

Kristin Eden

How would you explain the main findings of your paper to non-scientific family and friends?

When people think of allergies, they tend to think mostly of a runny nose or severe food allergies that lead to anaphylaxis. However, not very many people think about the mechanism underlying these symptoms. Likewise, people rarely consider other more chronic conditions, characterized by less acute symptoms that can affect the gut, especially since many of these diseases are relatively less common. My research focused on eosinophilic esophagitis (EoE), a chronic inflammatory disease that occurs in the upper gastrointestinal tract, which is basically inflammation in the throat that resembles an allergic response. Here, we identified a novel pathway that appears to impact symptoms of this disease. I found that the gene encoding a protein called NIK plays a protective role in EoE progression. Our findings show that mice that lack NIK develop an inflammatory disease of the esophagus, similar to human EoE, and that NIK may have importance in many cell types associated with the disease process.

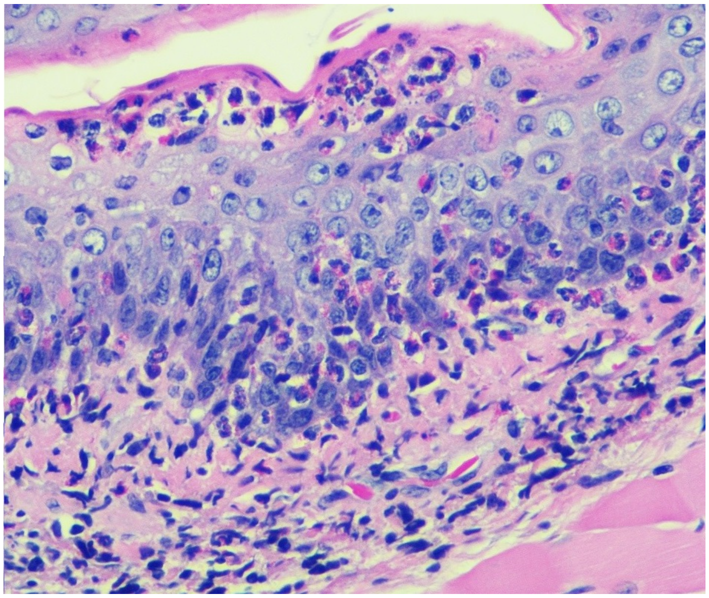

Eosinophils causing inflammation in the esophageal epithelium.

Kristin Eden's contact details: Department of Biomedical Sciences and Pathobiology, Virginia Maryland College of Veterinary Medicine, Blacksburg, VA 24060, USA.

E-mail: keden@vt.edu

What are the potential implications of these results for your field of research?

EoE is a relatively understudied disease. Thus, the model we describe in our recent work may be highly useful in evaluating new therapeutics and for further defining the mechanism/s underlying the disease in human patients. Likewise, the gene we have identified that encodes NIK has not been broadly characterized and it may also present a new therapeutic target that can affect both the immune system and the epithelial cells that line the esophagus. It was exciting to make a connection between a loss or disruption of NIK and a disease with human implications, as dyregulation of the pathway controlled by NIK has not been previously explored in eosinophilic diseases of the upper gastrointestinal system.

What has surprised you the most while conducting your research?

We were surprised to find this disease in our mice, given that our previous experience looking at the lower GI tract did not show the same phenotype. It was also surprising how localized the phenotype is, similar to the human EoE. We were not expecting to see such a targeted GI phenotype.

What are the main advantages and drawbacks of the model system you have used as it relates to the disease you are investigating?

While the model we describe is excellent for many aspects of disease pathogenesis, it is not perfect. Further work needs to be done to better define cell-type-specific effects and the timing of disease progression

and correlate these findings between the mouse and human. The global knockout animals used here are an informative stepping stone for determining the more specific effects of target genes – sort of like taking a large part out of a machine and then adding small pieces back bit by bit to determine the most essential cogs.

> "Proper mentoring is absolutely essential in early career development for scientists, and particularly career-oriented mentoring such as coaching for job searches and grant applications."

Describe what you think is the most significant challenge impacting your research at this time and how will this be addressed over the next 10 years?

I believe a significant challenge is the communication of people from various backgrounds and fields into projects. Of course, interdisciplinary research with varied faculty is highly sought after and encouraged, but there are of course always problems with communication, experience, and overall goals. As a veterinary pathologist, I need to make my experience and language accessible to basic science researchers, who in turn must be able to communicate with clinicians and ultimately patients. Multicomponent teams can be intensely efficient at creating data, but we must also be able to make sure our data is understood not only within our own groups, but with the downstream recipients of our work; including other scientists, doctors, and the general public.

What changes do you think could improve the professional lives of early-career scientists?

Proper mentoring is absolutely essential in early career development for scientists, and particularly career-oriented mentoring such as coaching for job searches and grant applications. Both of these processes are stumbling blocks, yet are critical for the success of investigators as they move towards independence.

What's next for you?

I'm relatively open with my career options. I would like to remain in academia, but have also developed an interest in both government and industry. As a DVM, and soon to be PhD, there are several opportunities for me in each sector and I look forward to exploring my options.

Reference

Eden, K., Rothschild, D. E., McDaniel, D. K., Heid, B. and Allen, I. C. (2017). Noncanonical NF-κB signaling and the essential kinase NIK modulate crucial features associated with eosinophilic esophagitis pathogenesis. *Dis. Model. Mech.* **10**, doi:10.1242/dmm.030767.
